# Comparison of the Genome-Wide DNA Methylation Profiles between Fast-Growing and Slow-Growing Broilers

**DOI:** 10.1371/journal.pone.0056411

**Published:** 2013-02-18

**Authors:** Yongsheng Hu, Haiping Xu, Zhenhui Li, Xuejuan Zheng, Xinzheng Jia, Qinghua Nie, Xiquan Zhang

**Affiliations:** 1 Department of Animal Genetics, Breeding and Reproduction, College of Animal Science, South China Agricultural University, Guangzhou, Guangdong, China; 2 Guangdong Provincial Key Lab of Agro-Animal Genomics and Molecular Breeding and Key Lab of Chicken Genetics, Breeding and Reproduction, Ministry of Agriculture, Guangzhou, Guangdong, China; University of Insubria, Italy

## Abstract

**Introduction:**

Growth traits are important in poultry production, however, little is known for its regulatory mechanism at epigenetic level. Therefore, in this study, we aim to compare DNA methylation profiles between fast- and slow-growing broilers in order to identify candidate genes for chicken growth. Methylated DNA immunoprecipitation-sequencing (MeDIP-seq) was used to investigate the genome-wide DNA methylation pattern in high and low tails of Recessive White Rock (WRR_h_; WRR_l_) and that of Xinhua Chickens (XH_h_; XH_l_) at 7 weeks of age. The results showed that the average methylation density was the lowest in CGIs followed by promoters. Within the gene body, the methylation density of introns was higher than that of UTRs and exons. Moreover, different methylation levels were observed in different repeat types with the highest in LINE/CR1. Methylated CGIs were prominently distributed in the intergenic regions and were enriched in the size ranging 200–300 bp. In total 13,294 methylated genes were found in four samples, including 4,085 differentially methylated genes of WRR_h_ Vs. WRR_l_, 5,599 of XH_h_ Vs. XH_l_, 4,204 of WRR_h_ Vs. XH_h_, as well as 7,301 of WRR_l_ Vs. XH_l_. Moreover, 132 differentially methylated genes related to growth and metabolism were observed in both inner contrasts (WRR_h_ Vs. WRR_l_ and XH_h_ Vs. XH_l_), whereas 129 differentially methylated genes related to growth and metabolism were found in both across-breed contrasts (WRR_h_ Vs. XH_h_ and WRR_l_ Vs. XH_l_). Further analysis showed that overall 75 genes exhibited altered DNA methylation in all four contrasts, which included some well-known growth factors of IGF1R, FGF12, FGF14, FGF18, FGFR2, and FGFR3. In addition, we validate the MeDIP-seq results by bisulfite sequencing in some regions.

**Conclusions:**

This study revealed the global DNA methylation pattern of chicken muscle, and identified candidate genes that potentially regulate muscle development at 7 weeks of age at methylation level.

## Introduction

Chicken growth is important economic traits in poultry production. It was determined by the interactions among genetic, nutritional, and environmental factors [1]. Until now, there have been extensive genome-wide association studies, which have identified some genetic factors affecting chicken growth [2], [3]. And many candidate genes were reported to have important effects on growth [4]–[6]. Moreover, a large number of quantitative trait loci (QTLs) for chicken growth have been identified [7]–[11]. However, the genetic mechanisms in chicken growth system are still unknown and, polymorphism or QTL alone can not provide adequate explanations for them. Recently, epigenetic factors especially DNA methylation have received considerable attention because of its potential influence on complex traits and diseases [12]. Nevertheless, so far the epigenetic mechanisms responsible for chicken growth remain poorly understood.

DNA methylation is a stably inherited epigenetic modification in eukaryotes. Previous work has demonstrated the importance of DNA methylation in many biological processes like gene expression regulation, genomic imprinting, X chromosome inactivation, and disease development [13]–[19]. Recently, the research on genomic methylation has been extensively conducted in plants and mammals [20]–[22]. In birds, the genome-wide DNA methylation was firstly profiled in the muscle and liver tissues from two breeds including the red jungle fowl and avian broiler using Methylated DNA immunoprecipitation-sequencing (MeDIP-seq) [23].

The objective of the present study was to assay the genome-wide DNA methylation pattern in the muscle and to identify methylated genes that were involved in the chicken growth. Here, we collected breast muscle tissues of the two-tail samples from two chicken breeds exhibiting different growth performance at 7 weeks of age: Recessive White Rock (WRR) and Xinhua Chickens (XH), and compared the DNA methylation differences between these two breeds and within each breed by MeDIP-seq. Our analysis showed the landscape of DNA methylome distribution in the genome, revealed a large number of differentially methylated genes in different comparisons between or within two breeds, and identified genes related to the regulation of chicken growth at 7 weeks of age.

## Materials and Methods

### Ethics Statement

All animal experiments were handled in compliance with and approved by the Animal Care Committee of South China Agricultural University (Guangzhou, People’s Republic of China) with approval number SCAU#0011. All efforts were made to minimize suffering.

### Animals

Two chicken breeds, WRR and XH, were used for DNA methylation investigation in the present study. WRR, a breed with fast growth rate, were obtained from Guangdong Wens Foodstuff Company Ltd, Guangdong, China. XH, a Chinese native breed with slow growth rate, were obtained from Zhicheng Avian Breeding Company Ltd, Guangdong, China. All broilers were reared in cages with a 24-h photoperiod for the first 2 d of age and then changed to a 16-h photoperiod. They were fed with free access to water and fed *ad libitum* with 16.5% CP and 2, 800 kcal of ME/kg. At 7 weeks of age, according to the body weight records, 3 female birds from each of the two-tail samples of WRR and XH were selected and then four groups including WRR_h_, WRR_l_, XH_h_, and XH_l_ were generated. The BW values were 1,064.0±11.1, 695.0±24.4, 305.8±23.3, and 207.6±11.1 g in the WRR_h_, WRR_l_, XH_h_, and XH_l_ group, respectively. Breast muscle tissues of the 12 individuals were collected and stored at −80°C until DNA extraction.

### DNA Extraction and Preparation for MeDIP-seq

Genomic DNA was isolated using TaKaRa Universal Genomic DNA Extraction Kit Ver. 3.0 (DV811A) (TaKaRa, Osaka, Japan) according to the manufacturer’s protocol and then DNA quality was evaluated by agarose gel electrophoresis and spectrophotometer. DNA from 3 birds within each group was mixed in equal amounts to generate a pooled sample using Quant-iT dsDNA HS Assay Kit (Invitrogen, Carlsbad, CA, USA). Subsequently, these four pooled samples were sonicated to produce DNA fragments ranging from 100–500 bp. After end repairing, phosphorylating and A-tailing with Paired-End DNA Sample Prep kit (Illumina, San Diego, CA, USA), DNA was ligated to an Illumina sequencing primer adaptor. Then the fragments were used for MeDIP enrichment using Magnetic Methylated DNA Immunoprecipitation kit (Diagenod, Liège, Belgium) following the manufacturer’s recommendation and the qualifying DNA was used for PCR amplification. Then bands between 220 and 320 bp were excised from the gel and purified with QIAquick Gel Extraction Kit (Qiagen, Valencia, CA, USA). Products were quantified with Quant-iTTM dsDNA HS Assay Kit (Invitrogen, Carlsbad, CA, USA) on an Agilent 2100 Analyzer (Agilent Technologies, Santa Clara, CA, USA). Following qPCR qualification, DNA libraries were sequenced on the Illumina Hiseq 2000 (Illumina, San Diego, CA, USA) to generate paired-end 50-bp reads by the Beijing Genomics Institute (BGI, Shenzhen, Guangdong, China).

### Bisulfite Sequencing

Five pairs of primers (Table 1) were designed with Methyl Primer Express Software v1.0, including one pair (P1) for the validation of relatively low methylated regions and four pairs (P2–P5) for high methylated regions. Two micrograms of pooled DNA from each group was firstly treated with the EpiTect Bisulfite kit (Qiagen, Valencia, CA, USA) and used as the template for the following semi-nested PCR amplification. PCR for PM1 and PM2 was performed in 50-µL reaction mixtures containing 50 ng of DNA, 1 µM of each primer and 25 µL Premix EX Taq™ Hot Start Version (TaKaRa, Osaka, Japan) with the conditions as: 94°C for 1 min; 35 cycles of 98°C for 10 s, 62°C for 30 s and 72°C for 30 s; and 72°C for 5 min. Reactions for PM3 to PM5 were carried out in a total volume of 50 µL including 50 ng of DNA, 1 µM of each primer and 2.5 U LA Taq HS (TaKaRa, Osaka, Japan). Both of the first and the second reaction rounds were performed under the following conditions: 94°C for 3 min; 35 cycles of 94°C for 30 s, 62°C for 30 s and 72°C for 30 s; and 72°C for 5 min. The PCR products were purified with a Gel Extraction Kit (Tiangen, Beijing, China) according to the manufacturer’s instructions and then cloned into the pMD18-T vector (Takara, Osaka, Japan). For each primer, 10 clones were sequenced by BGI (Shenzhen, Guangdong, China) with commercial service and the resulting data were analyzed using ClustalW.

**Table 1:** The information of primers for bisulfite sequencing.

Primers	Primer sequence (5′→3′)	Length^1^ (bp)	AT^2^ (°C)	Location^3^
PM1	F: GGTGGTAGTTGTATTTTTTTTGT	415	62	chr9: 6199130–6199544
	R: CTATACACAACTCCCCTAAACATA			
PM2	F:TTGATTGTAGTGGATTTGGATT	354	62	chr6: 10360074–10360427
	R: TACTCTCCTTCCAAACAAACC			
PM3	F: GGTTTGTTTGGAAGGAGAGTAA	357	62	chr6: 1036407–10360763
	R: AAAAAACCTCTACTCCACCTCC			
PM4	F: AGTAGGGGTGGATTTGGAATAT	346	62	chrUn_ Random: 45286930–45287275
	R: CAATCTTCCCTTCCCTAAAACT			
PM5	F: GTGAGTAGTTTTAGGGAAGGGA	433	62	chrUn_Random: 45287248–45287680
	R: ACTCCACCCCTACAAACTAAAC			

1 referred to the product length.

2 indicated annealing temperature.

3 indicated the PCR amplified locations in chicken chromosomes.

### Bioinformatic Analysis

Raw data obtained from Illumina sequencing were first processed to filter out reads containing adapters, unknown or low quality bases and then were mapped to the chicken reference genome (ftp://ftp.ensembl.org/pub/release-63/fasta/gallus_gallus/dna/) by SOAPaligner v 2.21 (http://soap.genomics.org.cn/) with no more than 2 bp mismatches [24]. The uniquely mapped data were retained for reads distribution analysis including the distribution in chicken chromosomes and the distribution in different components of the genome. Gene information was downloaded from the public FTP site of Ensembl (ftp://ftp.ensembl.org/pub/release-63/gtf/gallus_gallus/) and the region from transcript starting site to transcript ending site was defined as gene body region. The CpG islands (CGIs) were scanned by CpGPlot (https://gcg.gwdg.de/emboss/cpgplot.html) with the criteria as: length exceeding 200 bp, GC content greater than 50%, and observed-to-expected CpG ratio greater than 0.6. Repeat annotations were obtained from the UCSC database (http://hgdownload.cse.ucsc.edu/goldenPath/rn4/bigZips/chromOut.tar.gz) and the analysis of reads distribution on repeats was carried out by RepeatMasker (http://www.repeatmasker.org/). Then genome-wide methylation peak scanning was conducted using the MACS V 1.4.2 (http://liulab.dfci.harvard.edu/MACS/) [25]. The number of peaks in different components of the chicken genome (such as promoters, 5′ UTR, 3′ UTR, exon, intron, intergenic regions, CGIs, and repeats) was analyzed in our study. Moreover, the number of methylated peaks in the whole genome, called total peak number, was also analyzed in each sample and here a peak overlapping among the different components was just counted for one time. The methylation densities in different components of the genome were compared by calculating the ratio of methylated peaks in a particular component to the total area of that region. Statistical analyses of methylation level differences in different components of the genome and CGIs density differences in different size classes were processed with least square method by JMP 8.0 software (http://www.jmp.com/; SAS Institute Inc., Cary, NC, USA). All genes with peaks were used for the subsequent gene ontology (GO) analysis and pathway analysis. GO term information was obtained from the UniProtKB-GOA database (http://www.ebi.ac.uk/GOA/). Genes exhibiting more than 2-fold methylation level changes in different samples were analyzed for GO and KEGG pathway enrichments using the DAVID Functional Annotation Tool (http://david.abcc.ncifcrf.gov/) [26], with P<0.005 and Benjiamini adjusted p<0.05.

### Online Data Deposition

The MeDIP-Seq data from this study have been deposited in NCBI Sequence Read Archive with accession number GSE42751 (http://www.ncbi.nlm.nih.gov/geo/query/acc.cgi?acc=GSE42751).

## Results

### Assemble and Blast Analysis of MeDIP-seq Reads

In the present study, three breast muscle tissues were used to generate one pooled DNA sample for each group of WRR_h_, WRR_l_, XH_h_, and XH_l_. A range of 36,734,694 to 33,399,566 raw reads were generated for the four groups, respectively. In each group, about 65% of the reads were mapped and about 36% of the reads were uniquely mapped to the chicken genome (Table 2). The uniquely mapping reads of WRR_h_, WRR_l_, XH_h_, and XH_l_ covered 21.05%, 18.10%, 21.26%, and 20.03% of the chicken genome, respectively.

**Table 2:** Data generated by MeDIP-seq.

Sample^1^	Total numberof reads	Total MappedReads	Total Unique MappedReads	Percentage of mappedreads in total reads	Percentage of unique mapped reads
WRR_h_	36,734,694	23,877,624	13,087,223	65.00%	35.63%
WRR_l_	33,399,566	21,861,843	12,287,910	65.46%	36.79%
XH_h_	36,734,694	23,472,733	12,875,987	63.90%	35.05%
XH_l_	36,734,694	23,897,397	13,728,925	65.05%	37.37%

1 WRR_h_, WRR_l_, XH_h_, and XH_l_ indicated the group of Recessive White Rock with high body weight, Recessive White Rock with low body weight, Xinhua Chickens with high body weight, and Xinhua Chickens with low body weight, respectively.

MeDIP-seq reads were detected in most chromosomal regions (GGA1-28, chromosome Z, chromosome W, and chromosome MT) in each group except for some gaps (Figure S1, S2). However, no uniquely mapped but just multi-mapped reads could be found in a long region of GGA17 (from 3,180,001 to 11,182,526 bp).

**Figure 1:**
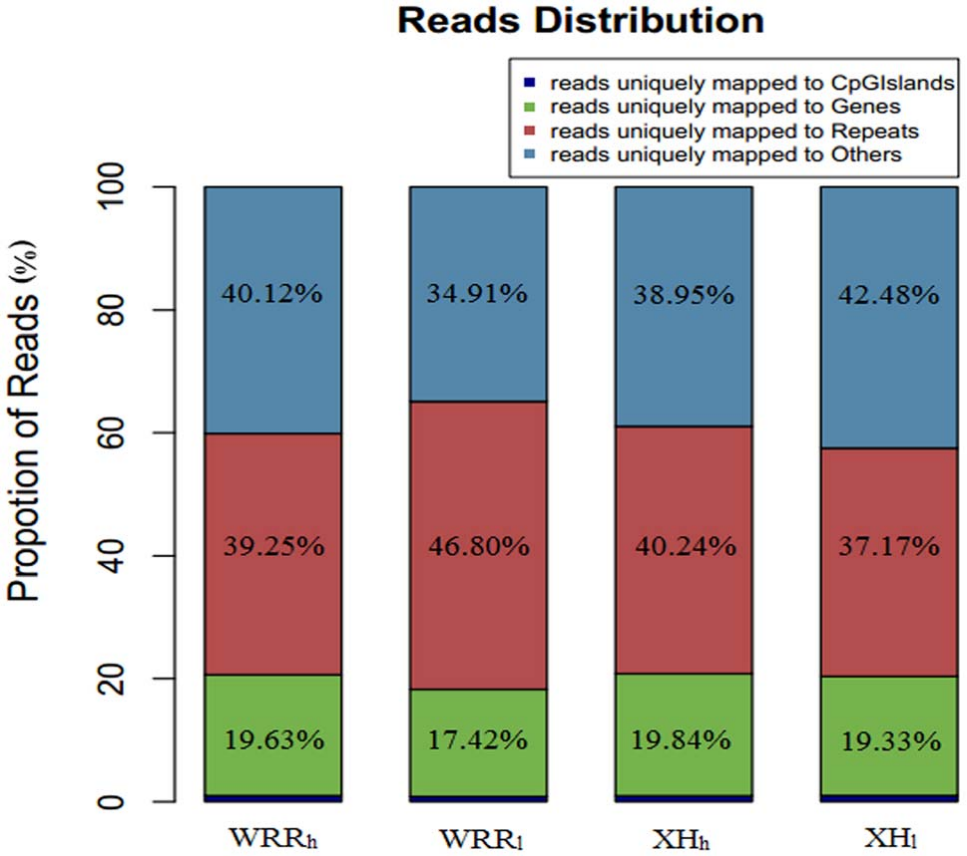
Genomic distribution of the uniquely mapped reads. All uniquely mapped reads were classified into four types: reads uniquely mapped to CpG islands (dark blue), genes bodies (green), repeats (red), others (light blue). The percentage for each class was given at the top of each graph. WRR_h_, WRR_l_, XH_h_, and XH_l_ indicated the group of Recessive White Rock with high body weight, Recessive White Rock with low body weight, Xinhua Chickens with high body weight, and Xinhua Chickens with low body weight, respectively.

The analysis of read distribution in different components of the genome showed that the uniquely mapped reads were mainly present in repeat elements. A range of 17.42% to 19.84% of them belonged to the gene body regions. The proportion of reads uniquely mapped to CGIs in WRR_h_, WRR_l_, XH_h_, and XH_l_ was only 1.00%, 0.87%, 0.97%, and 1.02%, respectively (Figure 1).

### MeDIP-seq Data Validation

**Figure 2:**
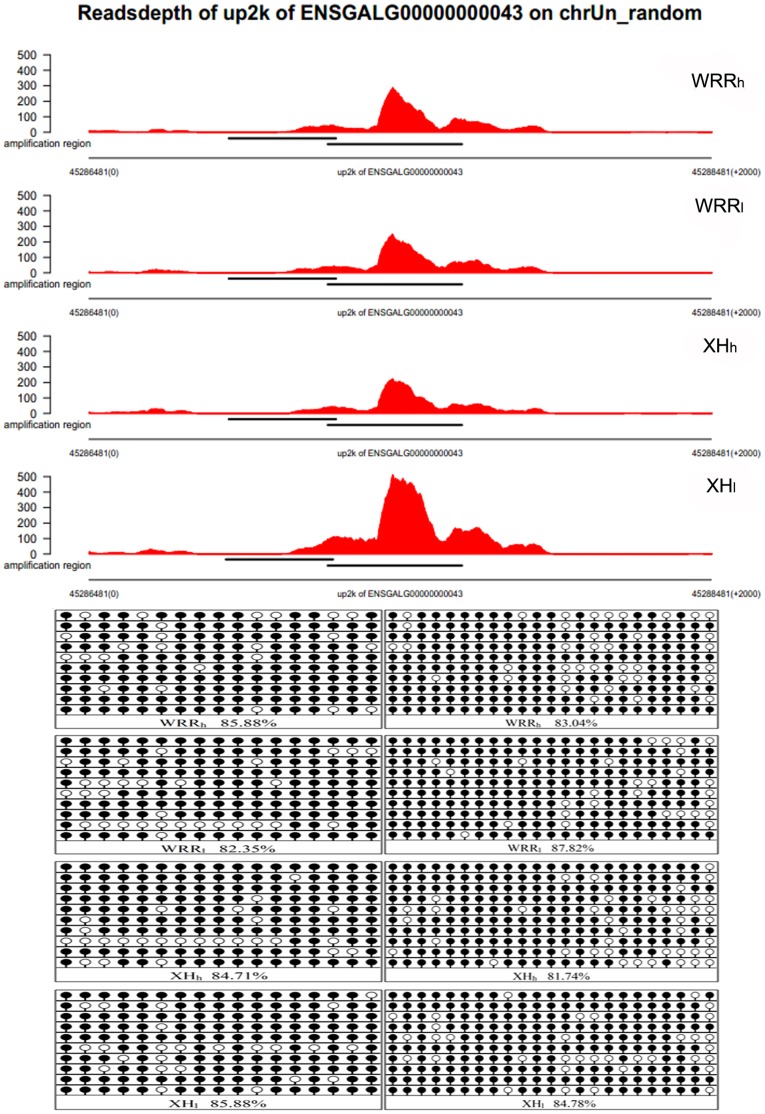
The validation of MeDIP-seq data by bisulfite sequencing. One region with high methylation obtained from MeDIP-Seq data was selected and its methylation pattern was assessed by bisulfite sequencing. Each line corresponded to a single strand of DNA and each circle represented a single CpG dinucleotide. Filled circles and open circles indicated methylated sites and unmethylated sites, respectively.

In this study, one region with relatively low methylation and two regions with high methylation were selected randomly to carry out bisulfite sequencing for the validation of MeDIP-seq data. We found that the bisulfite sequencing results were almost in accordance with the MeDIP-seq results (Figure 2, Figure S3 and S4).

### DNA Methylation Profiles of the Chicken

**Figure 3:**
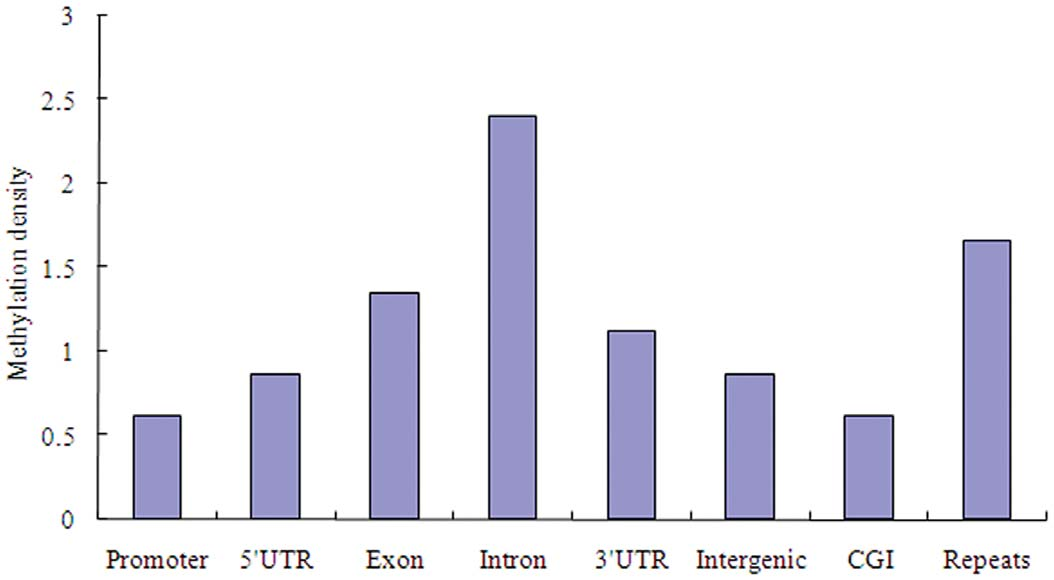
Methylation distribution in different genomic regions. Methylation density within promoter, gene body and intergenic regions was calculated with the ratio of methylated peaks in a particular component to the total area of that region.

In order to decipher the genome-wide DNA methylation profiles of the chicken, we used the uniquely mapped reads to detect the methylated peak and further analyzed the peak distribution in different components of the genome through the comparison of their methylation densities. Here, the genomic regions 2 Kb upstream and downstream of the TSS were regarded as the proximal promoter. We obtained 44,945, 44,832, 42,747, and 53,821 methylated peaks in WRR_h_, WRR_l_, XH_h_, and XH_l_, respectively (Table 3). A major portion of them were present in the intergenic regions followed by introns and exons. The average methylation density comparison showed that there were significantly differential methylation levels in different components of the genome (P<0.01) (Figure 3). Among all the classes, the average methylation density of promoters was the lowest followed by CGIs. The exon and intron regions exhibited significantly higher methylation levels than the intergenic regions (P<0.01). Within the gene body, the methylation density of introns was significantly higher than UTRs and exons (P<0.01). Repeats showed a relatively high methylation level. Moreover, we observed different methylation levels in different repeat types with high methylation in LINE/CR1 (44.5%), LTR/ERVL (20.6%), and simple repeat (9.3%) (Table 4).

**Table 3:** The peak distribution in different components of the chicken genome.

Sample^1^	Total peak number^2^	Promoter	5′UTR	Exon	Intron	3′UTR	Intergenic	CGI	Repeats
WRR_h_	44945	3838	608	10633	17689	1362	29390	4406	7493
WRR_l_	44832	3582	537	10388	17593	1268	31712	4020	6239
XH_h_	42747	3930	554	9970	16510	1278	27270	4412	6995
XH_l_	53821	4185	740	12781	20746	1563	36962	5084	7239

1 WRR_h_, WRR_l_, XH_h_, and XH_l_ indicated the group of Recessive White Rock with high body weight, Recessive White Rock with low body weight, Xinhua Chickens with high body weight, and Xinhua Chickens with low body weight, respectively.

2 Total peak number indicated the number of methylated peaks in the whole genome in each sample.

**Table 4:** The distribution of methylated peaks in different repeat types.

Repeat type	WRR_h_ 1	WRR_l_ 1	XH_h_ 1	XH_l_ 1
DNA	2.19	2.85	2.37	3.38
DNA/TcMar	0.87	1.09	0.81	1.22
LINE/CR1	44.57	43.23	41.39	48.78
Low_complexity	6.7	8.74	9.96	6.87
LTR	0.39	0.38	0.31	0.41
LTR/ERV1	3.88	2.48	2.56	2.69
LTR/ERVK	4.55	3.27	3.65	3.18
LTR/ERVL	21.83	21.16	20.24	19.34
rRNA	0.08	0.08	0.09	0.12
Satellite	2.72	3.14	4.15	3.34
Satellite/macro	2.88	1.28	0.96	1.41
Satellite/W-chromosome	1.07	1.23	1.16	1.11
Simple_repeat	7.79	10.34	11.68	7.39
SINE	0.19	0.3	0.29	0.43
tRNA	0.05	0.1	0.07	0.07
Unknown	0.23	0.32	0.31	0.26

1 WRR_h_, WRR_l_, XH_h_, and XH_l_ indicated the group of Recessive White Rock with high body weight, Recessive White Rock with low body weight, Xinhua Chickens with high body weight, and Xinhua Chickens with low body weight, respectively.

### Distribution of DNA Methylation in CGIs

**Figure 4:**
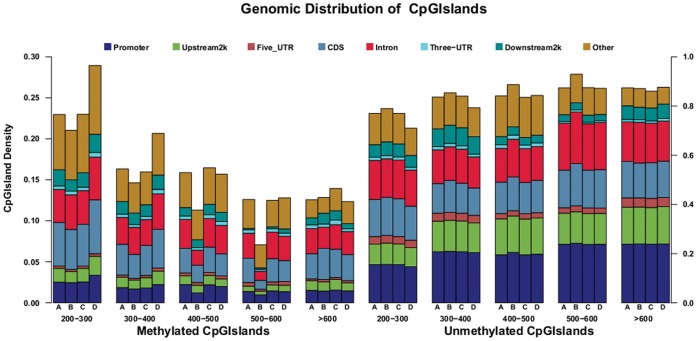
Genomic distribution of methylated and unmethylated CpG islands. We subdivided CpG islands into methylated and unmethylated islands and then categorized them into different bins according to their sizes. A. Genomic distribution of methylated CpG islands. B. Genomic distribution of unmethylated CpG islands. The number of CpG islands in a particular bin was calculated in different regions and subsequently it was normalized by the total number of CpG islands in that bin. Here the genomic region 2 kb upstream and downstream of the transcription start site was regarded as promoter. A, B, C, and D indicated the group of Recessive White Rock with high body weight (WRR_h_), Recessive White Rock with low body weight (WRR_l_), Xinhua Chickens with high body weight (XH_h_), and Xinhua Chickens with low body weight (XH_l_), respectively.

CGIs were associated with the majority of the annotated gene promoters and were reported to be lowly methylated in the vertebrate genome [27], [28]. In this study, CGIs were classified into two types based on their methylation status. CGIs containing methylated peaks were regarded as methylated CGIs and the rest were termed as unmethylated. In the chicken genome, there were a total of 33,915 CGIs. Of these CGIs, about 13.0% (n = 4,406) were methylated in WRR_h_, 11.9% (n = 4,020) in WRR_l_, 13.0% (n = 4,412) in XH_h_, and 15.0% (n = 5,084) in XH_l_ (Table 5). Most of the methylated CGIs were present in the intergenic regions. Within the gene body, exons showed more methylated CGIs than UTRs and introns. Moreover, when classified methylated CGIs of each class according to their sizes, we found that the CGI number significantly decreased (P<0.05) with increase in the size of islands except for that in the 3′UTR region and more than 20% of methylated CGIs were in the size range of 200–300 bp (Figure 4). The number of unmethylated CGIs was significantly more (P<0.01) than that of methylated CGIs in each size. The densities of unmethylated CGIs in different size classes were significantly different (P<0.05) for each region. Furthermore, we found that unmethylated CGIs were enriched in promoters compared to other classes (25%).

**Table 5:** Summary of methylated CGIs in the group of WRR_h_, WRR_l_, XH_h_, and XH_l_.

Sample^1^	5′UTR	3′UTR	Exon	Intron	Intergenic	Total methylated CGIs	Total CGIs	Methylated (%)
WRR_h_	54	88	1154	844	3195	4406	33915	13.0
WRR_l_	49	80	1044	750	2853	4020	33915	11.9
XH_h_	56	96	1158	838	3208	4412	33915	13.0
XH_l_	66	101	1322	970	3687	5084	33915	15.0

1 WRR_h_, WRR_l_, XH_h_, and XH_l_ indicated the group of Recessive White Rock with high body weight, Recessive White Rock with low body weight, Xinhua Chickens with high body weight, and Xinhua Chickens with low body weight, respectively.

### GO Analysis of Methylated Genes in the Four Samples

**Figure 5:**
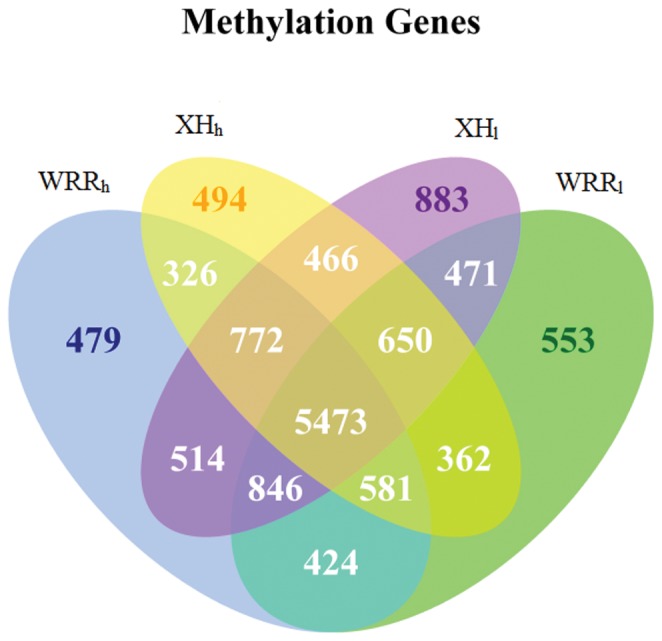
Methylated genes among four groups of WRR_h_, WRR_l_, XH_h_, and XH_l_. The methylated gene number was given at the top of each figure section. WRR_h_, WRR_l_, XH_h_, and XH_l_ indicated the group of Recessive White Rock with high body weight, Recessive White Rock with low body weight, Xinhua Chickens with high body weight, and Xinhua Chickens with low body weight, respectively.

**Figure 6:**
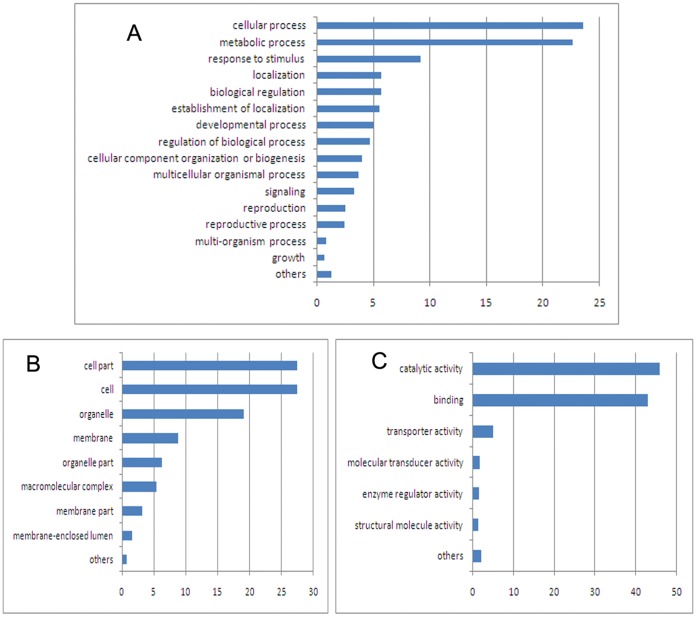
Functional classification of the whole methylated genes. (A) GO: Biological process. (B) Cellular component. (C) GO: Molecular function.

In the present study, genes that overlapped with the methylation peaks in promoters or gene body regions were termed as methylated genes. A total of 13,294 methylated genes were found in the four samples, including 9,415 in WRR_h_, 9,360 in WRR_l_, 9,124 in XH_h_, and 10,075 in XH_l_ (Figure 5). Of them, 5,473 methylated genes were identified in all of the four groups. GO assignments showed that these methylated genes were involved in one or more of the three categories: biological process, cellular component, and molecular function (Table S1, Dataset S1). Among them, 2,163 belonged to biological process categories, including cellular process (1,776; 23.55%), metabolic process (1,703; 22.58%), response to stimulus (690; 9.15%), localization (428; 5.67%), biological regulation (425; 5.64%), establishment of localization (416; 5.52%), and others (Figure 6A). Furthermore, 2,064 methylated genes belonged to cellular component categories, including cell part (2,025; 27.49%), cell (2,025; 27.49%), organelle (1,403; 19.05%), membrane (651; 8.84%), organelle part (457; 6.21%), macromolecular complex (399; 5.42%), membrane part (237; 3.22%), membrane-enclosed lumen (114; 1.55%), and others (54; 0.73%) (Figure 6B). On the other hand, a total of 2,471 methylated genes were found to be involved in molecular function categories, including catalytic activity (1,992; 45.73%), binding (1,868; 42.89%), transporter activity (218; 5.00%), molecular transducer activity (75; 1.72%), enzyme regulator activity (60; 1.38%), structural molecule activity (58; 1.33%), and others (85; 1.95%) (Figure 6C).

### Differentially Methylated Genes Among the Four Samples

**Figure 7:**
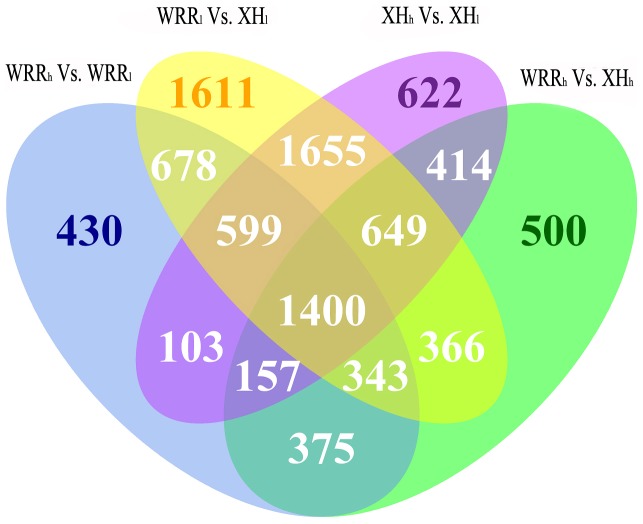
Differentially methylated genes unique or shared among four contrasts of WRR_h_ Vs. WRR_l_, XH_h_ Vs. XH_l,_ WRR_h_ Vs. XH_h,_ and WRR_l_ Vs. XH_l_. The number of differently methylated genes in each comparison was given at the top of each section of figures. WRR_h_ Vs. WRR_l_ indicated the comparison between the two-tail samples of Recessive White Rock. XH_h_ Vs. XH_l_ indicated the comparison between the two-tail samples of Xinhua Chickens. WRR_h_ Vs. XH_h_ indicated the comparison between the groups of Recessive White Rock and Xinhua Chickens with high body weight. WRR_l_ Vs. XH_l_ indicated the comparison between the groups of Recessive White Rock and Xinhua Chickens with low body weight.

Comparison of gene methylation showed that there were 4,085 differentially methylated genes (coverage changes was more than two folds; p value <0.01) between WRR_h_ and WRR_l_ (WRR_h_ Vs. WRR_l_), 5,599 between XH_h_ and XH_l_ (XH_h_ Vs. XH_l_), 4,204 between WRR_h_ and XH_h_ (WRR_h_ Vs. XH_h_), as well as 7,301 between WRR_l_ and XH_l_ (WRR_l_ Vs. XH_l_) (Figure 7, Dataset S2). Moreover, 2,259 differentially methylated genes were found in both WRR_h_ Vs. WRR_l_ and XH_h_ Vs. XH_l_, while 2,758 were identified in both WRR_h_ Vs. XH_h_ and WRR_l_ Vs. XH_l_. Of these, 1,400 genes were differently methylated in all of the four comparisons. We subsequently analyzed the direction and degree of methylation difference for the four contrasts in different gene regions. The results showed that there were more down-methylated genes than up-methylated genes in the WRR_h_ Vs. WRR_l_ and WRR_h_ Vs. XH_h_ contrasts, whereas a greater number of up-methylated than down-methylated genes were observed in both XH_h_ Vs. XH_l_ and WRR_l_ Vs. XH_l_ (Table 6). Furthermore, there were 12, 3, 151, 562, 4, and 7 common differentially methylated genes between WRR_h_ Vs. WRR_l_ (up) and XH_h_ Vs. Xh_l_ (up) in the upstream 2 k, 5′ UTR, exon, intron, 3′ UTR, and downstream 2 k, respectively, and 5, 0, 74, 528, 2, and 3 common genes between WRR_h_ Vs. WRR_l_ (down) and XH_h_ Vs. Xh_l_ (down) in those regions, respectively (Table S2). On the other hand, 56, 26, 332, 947, 15, and 45 common genes were found between WRR_h_ Vs. XH_h_ (up) and WRR_l_ Vs. Xh_l_ (up) in the upstream 2 k, 5′ UTR, exon, intron, 3′ UTR, and downstream 2 k, respectively, and 13, 3, 113, 570, 1, and 18 common genes between WRR_h_ Vs. XH_h_ (down) and WRR_l_ Vs. Xh_l_ (down) in those regions, respectively (Table S2).

**Table 6:** Numbers of differentially methylated genes for each contrast in different gene regions.

Contrast^1^	Upstream 2 k	5′UTR	Exon	Intron	3′UTR	Downstream 2 k
WRR_h_ Vs. WRR_l_ (up)	108	20	474	1135	32	89
WRR_h_ Vs. WRR_l_ (down)	367	71	1447	2396	160	303
XH_h_ Vs. XH_l_ (up)	700	179	2665	3373	341	578
XH_h_ Vs. XH_l_ (down)	100	12	449	1198	33	84
WRR_h_ Vs. XH_h_ (up)	192	48	739	1571	57	161
WRR_h_ Vs. XH_h_ (down)	291	65	1187	2127	132	257
WRR_l_ Vs. XH_l_ (up)	1138	349	3830	4587	585	996
WRR_l_ Vs. XH_l_ (down)	115	16	468	1276	33	107

1 WRR_h_, WRR_l_, XH_h_, and XH_l_ indicated the group of Recessive White Rock with high body weight, Recessive White Rock with low body weight, Xinhua Chickens with high body weight, and Xinhua Chickens with low body weight, respectively. For each contrast, up meant that there were greater peaks in the second group than the first group within the same region, whereas down meant there were greater peaks in the first group than the second group (p value<0.01).

### KEGG Pathway Analysis

In order to investigate the pathway categories of differentially methylated genes, we performed a DAVID functional annotation analysis. The results showed that the common differentially methylated genes of the WRR_h_ Vs. WRR_l_ and XH_h_ Vs. XH_l_ contrasts were significantly enriched (Benjiamini adjusted p<0.05) in 9 predicted pathways, including several growth and metabolic related pathways such as Wnt signaling pathway, MAPK signaling pathway, ErbB signaling pathway, focal adhesion, and adherens junction (Table 7). A total of 132 differentially methylated genes involved in these 5 pathways were observed in the contrasts within the two breeds (WRR and XH) (Table S3) and some of those genes were crucial to chicken growth: IGF1, IGF1R, MYL9, MYLK, FGF12, FGF13, FGF14, FGF18, FGFR1, FGFR2, FGFR3, etc. Analysis of the common differentially methylated genes in the WRR_h_ Vs. XH_h_ and WRR_l_ Vs. XH_l_ contrasts showed significant enrichment (Benjiamini adjusted p<0.05) in 8 KEGG pathways, including some related to growth and metabolic such as MAPK signaling pathway, adherens junction, focal adhesion, and tight junction (Table 8). There were 129 differentially methylated genes in these 4 pathways, including some affecting growth such as IGF1R, MYH11, MYH15, MYH7B, MYLK2, FGF12, FGF14, FGF18, FGFR2, FGFR3, TGFBR1, and TGFBR2 (Table S4). Further analysis of differentially methylated genes in pathways we concerned showed that 75 genes exhibited altered DNA methylation in all of the four contrasts including WRR_h_ Vs. WRR_l_, XH_h_ Vs. XH_l_, WRR_h_ Vs. XH_h_, and WRR_l_ Vs. XH_l_ (Table 9). Moreover, IGF1R and several genes belonging to the FGF family and receptors (FGF12, FGF14, FGF18, FGFR2, and FGFR3) were contained among them.

**Table 7:** KEGG pathways in which the common differentially methylated genes of WRR_h_ Vs. WRR_l_ and XH_h_ Vs. XH_l_ enriched.

No.	Pathways	P value	Benjiamini^1^
1	Focal adhesion	4.60E−05	5.90E−03
2	Wnt signaling pathway	2.30E−04	1.50E−02
3	MAPK signaling pathway	2.80E−04	1.20E−02
4	Melanogenesis	3.10E−04	1.00E−02
5	ErbB signaling pathway	4.70E−04	1.20E−02
6	Vascular smooth muscle contraction	4.80E−04	1.00E−02
7	Phosphatidylinositol signaling system	1.40E−03	2.50E−02
8	Calcium signaling pathway	1.50E−03	2.50E−02
9	Adherens junction	2.60E−03	3.70E−02

1 KEGG pathway enrichments were performed with the DAVID Functional Annotation Tool (http://david.abcc.ncifcrf.gov/) and Benjiamini adjusted p<0.05 was regarded as enriched.

**Table 8:** KEGG pathways in which the common differentially methylated genes of WRR_h_ Vs. XH_h_ and WRR_l_ Vs. XH_l_ enriched.

No.	Pathways	P value	Benjiamini^1^
1	MAPK signaling pathway	3.00E−04	3.80E−02
2	Adherens junction	4.10E−04	2.60E−02
3	Focal adhesion	4.50E−04	1.90E−02
4	Melanogenesis	5.60E−04	1.80E−02
5	Tight junction	1.20E−03	3.00E−02
6	Phosphatidylinositol signaling system	1.40E−03	2.90E−02
7	Calcium signaling pathway	1.80E−03	3.20E−02
8	Vascular smooth muscle contraction	2.00E−03	3.20E−02

1 KEGG pathway enrichments were performed with the DAVID Functional Annotation Tool (http://david.abcc.ncifcrf.gov/) and Benjiamini adjusted p<0.05 was regarded as enriched.

**Table 9:** Differentially methylated genes shared by WRR_h_ Vs. WRR_l_, XH_h_ Vs. XH_l_, WRR_h_ Vs. XH_h_, and WRR_l_ Vs. XH_l_.

No.	Gene	Description
1	ACTN1	actinin, alpha 1
2	AKT3	v-akt murine thymoma viral oncogene homolog 3 (protein kinase B, gamma)
3	BCL2	B-cell CLL/lymphoma 2
4	CACNA1B	calcium channel, voltage-dependent, N type, alpha 1B subunit
5	CACNA1D	calcium channel, voltage-dependent, L type, alpha 1D subunit
6	CACNA1H	calcium channel, voltage-dependent, T type, alpha 1H subunit
7	CACNA1I	calcium channel, voltage-dependent, T type, alpha 1I subunit
8	CACNA2D1	calcium channel, voltage-dependent, alpha 2/delta subunit 1; similar to voltage-gated calcium channel alpha2/delta-1 subunit
9	CACNA2D3	calcium channel, voltage-dependent, alpha 2/delta 3 subunit
10	CACNB2	calcium channel, voltage-dependent, beta 2 subunit
11	CACNG2	calcium channel, voltage-dependent, gamma subunit 2
12	CAPN2	calpain 2, (m/II) large subunit
13	COL5A2	collagen, type V, alpha 2
14	COL6A2	collagen, type VI, alpha 2
15	CREBBP	CREB binding protein (Rubinstein-Taybi syndrome)
16	CSNK2A1	casein kinase 2, alpha 1 polypeptide
17	CTNNA2	catenin (cadherin-associated protein), alpha 2
18	CTNNA3	catenin (cadherin-associated protein), alpha 3
19	EP300	E1A binding protein p300
20	EVI1	ecotropic viral integration site 1
21	FARP2	FERM, RhoGEF and pleckstrin domain protein 2
22	FGF12	fibroblast growth factor 12
23	FGF14	fibroblast growth factor 14
24	FGF18	fibroblast growth factor 18
25	FGFR2	fibroblast growth factor receptor 2
26	FGFR3	fibroblast growth factor receptor 3
27	FLNB	filamin B, beta (actin binding protein 278)
28	FLT1	fms-related tyrosine kinase 1 (vascular endothelial growth factor/vascular permeability factor receptor)
29	GSK3B	glycogen synthase kinase 3 beta
30	HRAS	v-Ha-ras Harvey rat sarcoma viral oncogene homolog
31	IGF1R	insulin-like growth factor 1 receptor
32	ITGA9	integrin, alpha 9
33	ITGB1	integrin, beta 1 (fibronectin receptor, beta polypeptide, antigen CD29 includes MDF2, MSK12)
34	ITGB5	integrin, beta 5
35	KRAS	v-Ki-ras2 Kirsten rat sarcoma viral oncogene homolog
36	LAMA3	laminin, alpha 3
37	LAMB3	laminin, beta 3
38	LMO7	LIM domain 7
39	LOC422316	similar to receptor tyrosine kinase flk-1/VEGFR-2
40	MAP2K4	mitogen-activated protein kinase kinase 4
41	MAP2K5	mitogen-activated protein kinase kinase 5
42	MAP3K3	mitogen-activated protein kinase kinase kinase 3
43	MAP3K5	mitogen-activated protein kinase kinase kinase 5
44	MAP3K7	mitogen-activated protein kinase kinase kinase 7
45	MAP4K4	mitogen-activated protein kinase kinase kinase kinase 4; similar to mitogen-activated protein kinase kinase kinase kinase 4
46	MAPK14	mitogen-activated protein kinase 14
47	MAPKAPK2	mitogen-activated protein kinase-activated protein kinase 2
48	MAPKAPK5	mitogen-activated protein kinase-activated protein kinase 5
49	MKNK1	MAP kinase interacting serine/threonine kinase 1
50	NF1	neurofibromin 1
51	NFKB1	nuclear factor of kappa light polypeptide gene enhancer in B-cells 1
52	PAK7	p21(CDKN1A)-activated kinase 7
53	PARD3	par-3 partitioning defective 3 homolog (C. elegans)
54	PARVA	parvin, alpha
55	PARVB	parvin, beta
56	PDGFA	platelet-derived growth factor alpha polypeptide
57	PIK3CB	phosphoinositide-3-kinase, catalytic, beta polypeptide
58	PIK3R3	phosphoinositide-3-kinase, regulatory subunit 3 (p55, gamma)
59	PIK3R5	phosphoinositide-3-kinase, regulatory subunit 5, p101
60	PLA2G4A	phospholipase A2, group IVA (cytosolic, calcium-dependent)
61	PPP1R12A	protein phosphatase 1, regulatory (inhibitor) subunit 12A
62	PPP2CB	protein phosphatase 2 (formerly 2A), catalytic subunit, beta isoform
63	PPP3CB	protein phosphatase 3 (formerly 2B), catalytic subunit, beta isoform
64	PRKCA	protein kinase C, alpha
65	PTK2	PTK2 protein tyrosine kinase 2
66	PTPRR	protein tyrosine phosphatase, receptor type, R
67	RELN	reelin
68	RPS6KA2	ribosomal protein S6 kinase, 90kDa, polypeptide 2
69	SOS2	son of sevenless homolog 2 (Drosophila)
70	SSX2IP	synovial sarcoma, X breakpoint 2 interacting protein
71	TCF7	transcription factor 7 (T-cell specific, HMG-box)
72	TCF7L2	transcription factor 7-like 2 (T-cell specific, HMG-box)
73	VAV3	vav 3 oncogene
74	XIAP	X-linked inhibitor of apoptosis
75	YES1	v-yes-1 Yamaguchi sarcoma viral oncogene homolog 1

## Discussion

### DNA Methylation Profiles

Although global DNA methylation surveys have been performed on liver and muscle tissues [23], this study is the first to systematically compare the genome-wide muscle methylation profiles of fast- and slow-growing broilers using two-tail samples of two breeds with different growth performance. The objective was to identify methylated genes affecting chicken growth. In the present study, the MeDIP-seq method was applied and 4 lines were employed in all, each line using pooled DNA samples from 3 birds. Such a pooling strategy can reduce the cost. To confirm results from MeDIP-seq, methylation tests of three regions were done with bisulfite sequencing in each pooled samples. And the methylation levels between the two methods were generally in accord with each other. Reads distribution analysis of our study found that uniquely mapped reads were enriched in the repeats and the gene body regions. It was consistent with previous findings [23].

The scan of methylation enriched regions (called peak) in MeDIP-seq was important to survey the global methylation pattern. In this study, peak distribution analysis demonstrated that promoter and CGIs were hypomethylated, whereas the methylation levels in gene body regions and repeats were relatively high. These results were in accordance with findings in other species [22], [29]. It has been well documented that most of the promoter regions were lowly methylated and promoter DNA methylation had repressive effects on gene expression [30]. DNA methylation in the gene body regions might alter chromatin structure and transcription elongation efficiency [31]. However, in contrast to previous research in animals [22], [29], [32], we did not observed a higher methylation level in exons than in introns in chickens. Further analysis of the methylation levels in the gene body regions showed that there was no significant difference (P>0.05) among the methylation densities of the first exon (1.06±0.14), mid exon (1.43±0.14), last exon (1.23±0.14), and exons (1.34±0.14). Also no significant difference (P>0.05) was found among the methylation levels of the first intron (2.11±0.14), mid intron (2.32±0.14), last intron (2.55±0.14) and the intron region (2.39±0.14). On the other hand, it has been demonstrated that most of the CGIs were unmethylated and CGIs could influence local chromatin structure [33], [34]. Like the findings in the present study, the majority of methylated CGIs were observed in intragenic and intergenic regions [35], [36]. Intragenic or intergenic CGIs were proved to have the characteristics of functional promoters and the methylation of intragenic CGIs played a crucial role in regulating alternative promoters [34], [36], [37]. In chicken genome, the LINE/CR1 was the predominant interspersed repeat element and it accounted for over 80% of all interspersed repeats [38]. Our study here found that LINE/CR1 was the predominant repeats of DNA methylation, which was consistent with findings in previous study of chicken [23].

### Potential Pathways Involved in Chicken Growth at 7 Weeks of Age

Growth is under complex genetic control [39]. In the current study, in order to uncover its regulation mechanisms, the regulatory network underlying growth was examined. For those differentially methylated genes common for the contrasts compared within breeds or between breeds, enriched growth and metabolic related pathways were explored. As expected, several important pathways were found, including MAPK signaling pathway, Wnt signaling pathway, and ErbB signaling pathway. The MAPK signaling pathway is a well-known signal transduction pathway that can transduce a variety of external signals and subsequently lead to a wide range of cellular responses including growth, differentiation, inflammation and apoptosis. Currently, three major MAPK pathways, the extracellular-signal regulated kinases (ERK1/ERK2), the c-jun N-terminal kinases (JNK), and p38 kinase, have been identified [40]. Previous research showed that the MAPK (RAF/MEK/ERK) signaling pathway played a key role in skeletal muscle and its activation was indispensable for muscle cell proliferation [41]. And the p38 MAPK signaling pathway was proved to be a major regulator of skeletal muscle development [42]. On the other hand, the MAPK pathway is a common target downstream of all ErbB receptors, which are well-known mediators of cell proliferation, differentiation, apoptosis, and cell motility [43]. Thus, the ErbB signaling pathway was also selected as a possible pathway affecting growth in the present study. The Wnt signaling pathway was crucial for embryogenesis in vertebrates. In chicken, the Wnt signaling pathway was found to be strongly associated with some carcass traits [44]. In addition, our analyses also found some pathways related to cell junctions (tight junction, focal adhesion, adherens junction) enriched. Focal adhesion was the signaling center of numerous intracellular pathways that regulated cell growth, survival, and gene expression [45]. Moreover, recent studies suggested that the tight junction was involved in the regulation of cell growth and differentiation, while the adherens junction could limit cell growth [46]–[48]. Therefore, those three pathways were regarded as pathways potentially related to chicken growth at 7 weeks of age in this study.

### Function of Potential Methylated Genes Affecting Chicken Growth at 7 Weeks of Age

WRR and XH were two chicken breeds with divergent growth rate. In this study, the body weight of WRR was more than three times of the XH at seven weeks of age. Further, for the two-tail samples within each breed, the body weight of fast-growing samples was about 1.5 times more than slow-growing samples. Therefore, the identified differentially methylated genes within or between the two breeds in breast muscle tissues were potentially involved in chicken growth at 7 weeks of age. Eventually, we found that a total of 75 differentially methylated genes shared by all the four contrasts (WRR_h_ Vs. WRR_l_, XH_h_ Vs. XH_l,_ WRR_h_ Vs. XH_h,_ and WRR_l_ Vs. XH_l_) might contribute to the regulation of chicken growth at 7 weeks of age. Among them, IGF1R and several genes belonging to the FGF family and receptors (FGF12, FGF14, FGF18, FGFR2, and FGFR3) were contained. IGF1R has been well demonstrated to play an important role in the skeletal muscle development [49], [50]. In chicken, several polymorphisms of the IGF1R gene were identified to be associated with early growth traits and carcass traits [4]. FGFs were originally isolated as growth factors for fibroblasts, and now they were recognized as growth factors with diverse biological activities [51]. For instance, previous studies in rodents and chicken demonstrated that FGF18 was a pleiotropic growth factor involved in the development of various organs [52], [53]. Studies using FGF knockout mice also indicated that FGF18 played a crucial role in development [51]. FGFRs were also demonstrated to have crucial effects on cell proliferation [51]. The results from this study indicated that these genes might affect chicken growth at 7 weeks of age via the change of DNA methylation.

In addition, many other differentially methylated genes related to muscle development were found in both inner contrasts (WRR_h_ Vs. WRR_l_ and XH_h_ Vs. XH_l_), including the key modulator of skeletal muscle differentiation, IGF1 and well-known genes related to the biosynthesis of myosin (MYL9 and MYLK) [54]. The methylation of these genes might partially contribute to the chicken growth difference within breeds at 7 weeks of age. On the other hand, some well-known genes related to the biosynthesis of myosin (MYH11, MYH15, MYH7B, and MYLK2) and two genes essential for normal growth and development (TGFBR1 and TGFBR2) were observed in both across-breed contrasts (WRR_h_ Vs. XH_h_ and WRR_l_ Vs. XH_l_) [55], [56]. We believed that the methylation of these genes might partially contribute to the chicken growth difference between WRR and XH at 7 weeks of age. However, the epigenetic effects of these genes on chicken growth still require further study in the future.

In summary, this study provided a comprehensive analysis of DNA methylation profiles of chicken breast muscle and revealed 75 differentially methylated genes between fast- and slow-growing birds at 7 weeks of age. Several genes (IGF1R, FGF12, FGF14, FGF18, FGFR2, and FGFR3) may play key roles in affecting chicken growth at 7 weeks of age. Our observations provide new clues for deciphering the epigenetic mechanisms of chicken growth and will contribute to the improvement of poultry production.

## Supporting Information

Figure S1 **Chromosome distribution of reads in WRR_h_ and WRR_l_.** The distribution of reads in the chromosome 1–28, Z, W, and chromosome MT of the chicken genome was shown with red color for each sample. MeDIP-seq reads were plotted in 10 kb windows along chromosome. WRR_h_ and WRR_l_ indicated the group of Recessive White Rock with high body weight and Recessive White Rock with low body weight, respectively.
(JPG)

Figure S2 **Chromosome distribution of reads in XH_h_ and XH_l_.** The distribution of reads in the chromosome 1–28, Z, W, and chromosome MT of the chicken genome was shown with red color for each sample. MeDIP-seq reads were plotted in 10 kb windows along chromosome. XH_h_ and XH_l_ indicated the group of Xinhua Chickens with high body weight and Xinhua Chickens with low body weight, respectively.
(JPG)

Figure S3 **Bisulfite sequencing validation of MeDIP-seq data in one region with relatively low methylation.** WRR_h_, WRR_l_, XH_h_, and XH_l_ indicated the group of Recessive White Rock with high body weight, Recessive White Rock with low body weight, Xinhua Chickens with high body weight, and Xinhua Chickens with low body weight, respectively.
(JPG)

Figure S4 **Bisulfite sequencing validation of MeDIP-seq data in one region with relatively low methylation.** WRR_h_, WRR_l_, XH_h_, and XH_l_ indicated the group of Recessive White Rock with high body weight, Recessive White Rock with low body weight, Xinhua Chickens with high body weight, and Xinhua Chickens with low body weight, respectively.
(JPG)

Table S1 The GO categories of methylated genes.
(XLS)

Table S2 Differentially methylated genes overlapped among the four comparisons including WRR_h_ Vs.WRR_l_, WRR_h_ Vs.XH_h_, WRR_l_ Vs.XH_l_, and XH_h_ Vs.XH_l_ based on the up/down and gene body regions classifications.
(XLS)

Table S3 Differentially methylated genes related to chicken growth in both contrasts of WRR_h_ Vs. WRR_l_ and XH_h_ Vs. XH_l_.
(XLS)

Table S4 Differentially methylated genes related to chicken growth in both contrasts of WRR_h_ Vs. XH_h_ and WRR_l_ Vs. XH_l_.
(XLS)

Dataset S1 **The GO categories of methylated genes when genes were subdivided according to their methylated regions.**
(RAR)

Dataset S2 **Detail information of differentially methylated genes in the four comparisons including WRR_h_ Vs.WRR_l_, WRR_h_ Vs.XH_h_, WRR_l_ Vs.XH_l_, and XH_h_ Vs.XH_l_.**
(RAR)
