# Supplementary figures and images for: Comparison of the Genome-Wide DNA Methylation Profiles between Fast-Growing and Slow-Growing Broilers
Source: PLoS One. 2013 Feb 18;8(2):e56411. doi: 10.1371/journal.pone.0056411 (PMC3575439; doi:10.1371/journal.pone.0056411)

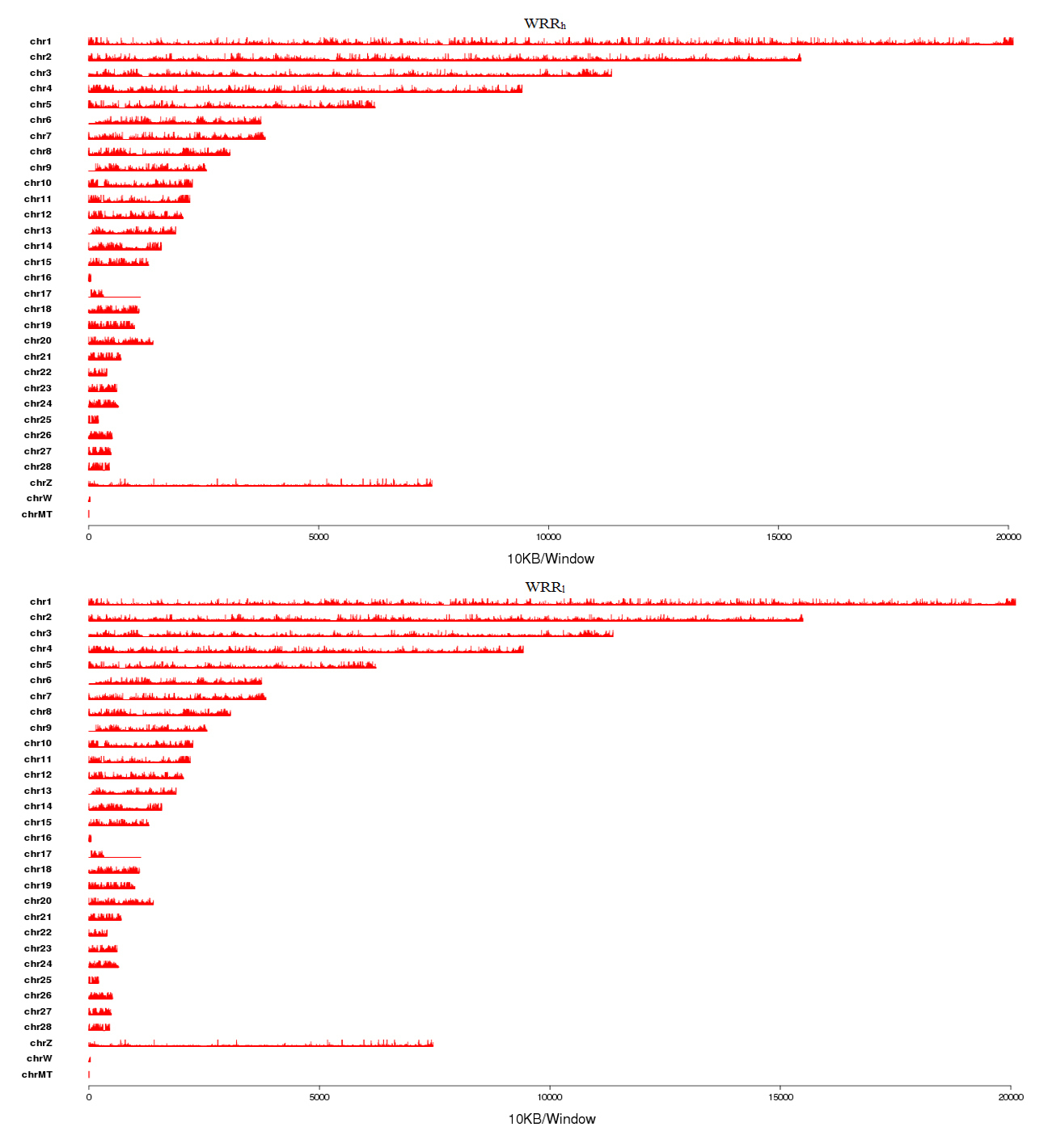

Supplement: Figure S1 — Chromosome distribution of reads in WRRh and WRRl. The distribution of reads in the chromosome 1–28, Z, W, and chromosome MT of the chicken genome was shown with red color for each sample. MeDIP-seq reads were plotted in 10 kb windows along chromosome. WRRh and WRRl indicated the group of Recessive White Rock with high body weight and Recessive White Rock with low body weight, respectively. (JPG) [file pone.0056411.s001.jpg]

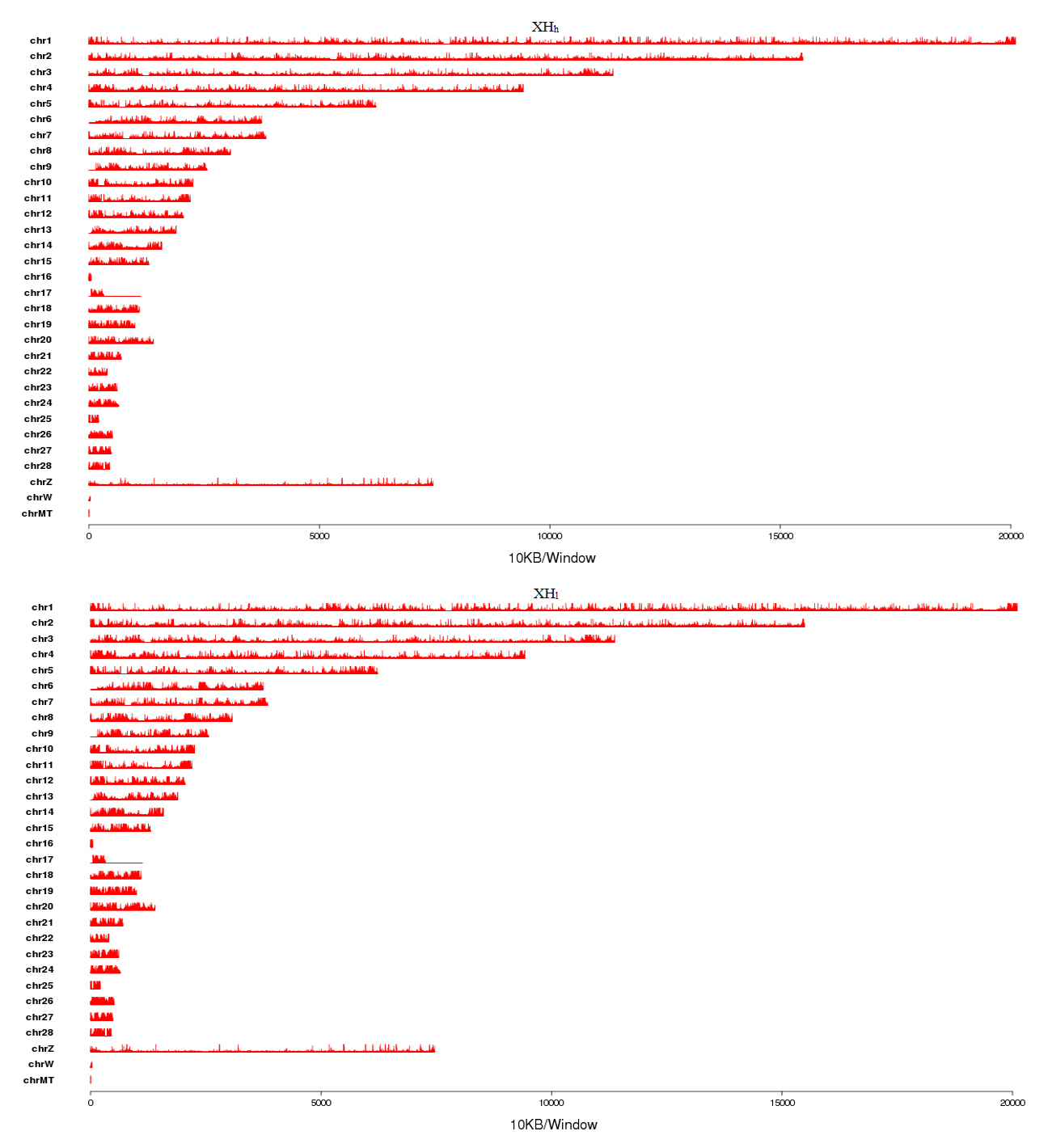

Supplement: Figure S2 — Chromosome distribution of reads in XHh and XHl. The distribution of reads in the chromosome 1–28, Z, W, and chromosome MT of the chicken genome was shown with red color for each sample. MeDIP-seq reads were plotted in 10 kb windows along chromosome. XHh and XHl indicated the group of Xinhua Chickens with high body weight and Xinhua Chickens with low body weight, respectively. (JPG) [file pone.0056411.s002.jpg]

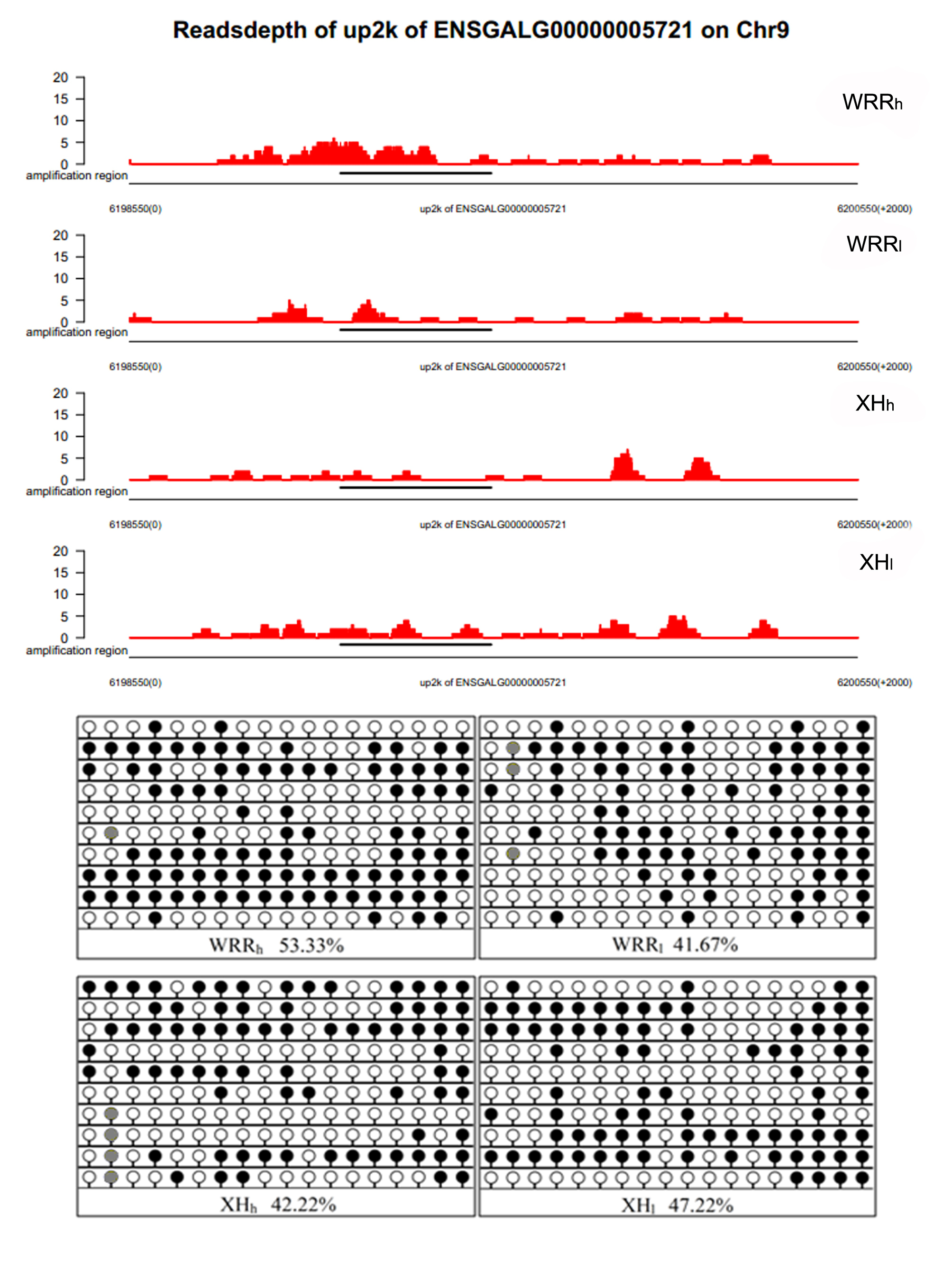

Supplement: Figure S3 — Bisulfite sequencing validation of MeDIP-seq data in one region with relatively low methylation. WRRh, WRRl, XHh, and XHl indicated the group of Recessive White Rock with high body weight, Recessive White Rock with low body weight, Xinhua Chickens with high body weight, and Xinhua Chickens with low body weight, respectively. (JPG) [file pone.0056411.s003.jpg]

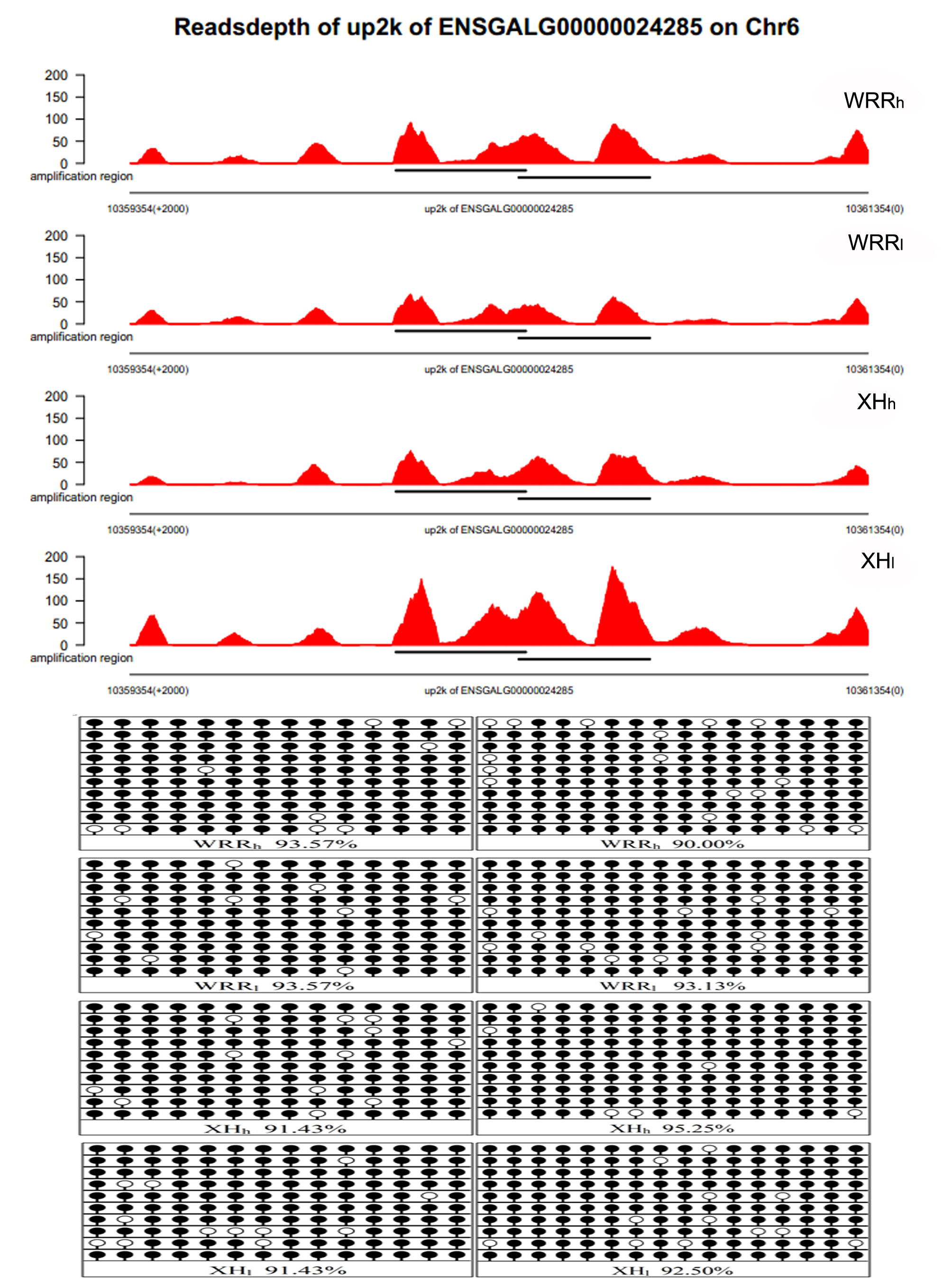

Supplement: Figure S4 — Bisulfite sequencing validation of MeDIP-seq data in one region with relatively low methylation. WRRh, WRRl, XHh, and XHl indicated the group of Recessive White Rock with high body weight, Recessive White Rock with low body weight, Xinhua Chickens with high body weight, and Xinhua Chickens with low body weight, respectively. (JPG) [file pone.0056411.s004.jpg]
